# Nanostructured Electrodes for High-Performance Supercapacitors and Batteries

**DOI:** 10.3390/nano13202807

**Published:** 2023-10-23

**Authors:** Xiang Wu

**Affiliations:** School of Materials Science and Engineering, Shenyang University of Technology, Shenyang 110870, China; wuxiang05@sut.edu.cn

Emerging renewable energy sources have received extensive attention in the past few decades. Energy storage has been one of the hottest research fields for over half a century. Recently, low-dimensional nanostructured materials have been widely investigated due to their fascinating performances in energy storage fields. Great efforts have been devoted to studying their synthesis strategies, unique properties, and potential applications in various electrochemical devices. Nevertheless, challenges still exist, and many energy devices are in high demand for practical applications. At the same time, the ever-increasing demand for alternative energy strategies to fossil fuels/electrochemical cells has initiated considerable efforts to develop novel and renewable energy storage systems. Considering the synergetic effects between different components, hybrid nanomaterials may demonstrate dramatically enhanced performance compared to their single components. Therefore, It is urgent and significant to have a Special Issue to appreciate updated advances and review recent progress regarding nanostructured electrodes for high-performance supercapacitors and batteries. In this Special Issue, we collected 21 papers, including 17 research articles and four review papers. It covers supercapacitors, sensors, catalysts, metal ion batteries, solar cells, etc.

Supercapacitors show an important development perspective owning to their high-power density and excellent cycling performance [1,2,3,4]. Obaidat et al. reported hierarchical CuMn_2_O_4_ nanosheet arrays nanostructures using a one-step hydrothermal route on a nickel foam substrate. The obtained samples were utilized as battery-type electrode material, which delivered a specific capacity of 125.56 mA h g^−1^ at 1 A g^−1^ with a rate capability of 84.1% and a cycling stability of 92.15% over 5000 cycles [5]. Niu and coworkers prepared α-Fe_2_O_3_@MnO_2_ electrode materials on carbon cloth using hydrothermal strategies and subsequent electrochemical deposition. The specific capacitance of the as-obtained product is 615 mF cm^−2^ at 2 mA cm^−2^. Moreover, a flexible supercapacitor presents an energy density of 0.102 mWh cm^−3^ at 4.2 W cm^−2^. Bending tests of the device at different angles show excellent mechanical flexibility [6]. Nitrogen/oxygen-doped porous carbon materials were also fabricated by calcining and activating an organic crosslinked polymer. The optimized porous carbon material showed a specific capacitance of 522 F g^−1^ at 0.5 A g^−1^ in a three-electrode system. Furthermore, an energy density of 18.04 Wh kg^−1^ was obtained at a power density of 200.0 W kg^−1^ in a two-electrode system [7]. NiMoO_4_ is a very suitable electrode material for SCs because of its advantages of outstanding electrochemical performance and low price. Zhao’s group synthesized NiMoO_4_@MnCo_2_O_4_ composite electrodes based on a two-step hydrothermal method. The sample reaches 3000 mF/cm^2^ at 1 mA/cm^2^. The asymmetric supercapacitor was constructed with activated carbon as the negative electrode, which showed a maximum energy density of 90.89 mWh/cm^3^ at a power density of 3726.7 mW/cm^3^, and the capacitance retention can achieve 78.4% after 10,000 cycles [8]. In addition, Svetukhin et al. prepared PANI/VA-MWCNT pseudo-capacitors and studied the device’s temperature-dependent charging–discharging dynamics [9]. Wang’s group summarized the research progress of cobalt-based nanomaterials as electrode materials for supercapacitors and focused on the strategies to improve the electrochemical properties of these materials [10]. Polyaniline (PANI) is thought to be an excellent candidate for energy-storage applications owing to its tunable structure, multiple oxidation/reduction reaction, and environmental stability. Scientists from different countries summarized updated progress about polyaniline/metal-organic framework composite electrodes for supercapacitor applications [11]. Zhang and coworkers fabricated the n*AlN/n*ScN superlattices by the epitaxial growth technology of controlling layer interface in an atom resolution. Their energy storage characteristics were studied using first principle calculation of the band-structure and dielectric polarizability dependent on the electrical field and superlattice configuration [12].

Besides the above supercapacitor reports, we collected some work about metal ion batteries, such as Li-ion, Zn ion, and sodium batteries. Zhang and You synthesized an environmentally friendly and cost-effective CoO@rGO flexible membrane with excellent electrochemical properties as anode material. It showed that the hollow material is much more favorable for lithium-ion transport and storage [13]. Cui et al. prepared fibrous red phosphorus as an anode material for LIBs using chemical vapor transport. The obtained composite material showed a reversible specific capacity of 1621 mAh/g and a capacity of 742.4 mAh/g after 700 cycles at a current density of 2 A/g. The Coulombic efficiencies reached almost 100% for each cycle [14]. It is known that Li-rich oxides are promising cathode materials for Li-ion batteries. Scientists from Russia studied different compositions of Li-rich materials and various electrochemical testing modes. The results showed that the Li_1.149_Ni_0.184_Mn_0.482_Co_0.184_O_2_ cathode material demonstrated the best functional properties [15]. SiO_2_ is also used as an anode materials for lithium battery. Qin et al. prepared SiO_2_ aerogels using a Sol-Gel method. The results showed that Ketjen Black provides superior cycling and rate performance with a reversible specific capacity of 351.4 mA h g^−1^ at 0.2 A g^−1^ after 200 cycles and 311.7 mA h g^−1^ at 1.0 A g^−1^ after 500 cycles [16]. Hierarchical Si@MnO_2_@reduced graphene oxide (rGO) can effectively reduce the volume change of Si and increase the lithium-ion battery capacity due to the dual protection of MnO_2_ and rGO. It showed a discharge-specific capacity of 1282.72 mAh g^−1^ at 1 A g^−1^ after 1000 cycles. Moreover, the volume expansion of the anode material is 50% after 150 cycles, which is much less than that of Si (300%) [17].

Aqueous zinc ion batteries (AZIBs) have attracted much attention owing to their low cost, high capacity, and non-toxic characteristics. Therefore, transition metal chalcogenides with a layered structure are considered suitable electrode materials. The large layer spacing facilitates the intercalation/de-intercalation of Zn^2+^ between the layers. Wu’s group summarized many design strategies for modifying cathodes and specifically emphasized the zinc storage capacity of the optimized electrodes. They also proposed the challenges and prospects of cathode materials for high-energy AZIBs [18]. Gong et al. investigated the PANI cathode’s electrochemical performance and ion transport kinetics to further understand Zn^2+^ storage mechanisms. The assembled PANI/Zn cell achieves a capacity of 74 mAh g^−1^ a t 0.3 A g^−1^ and maintains 48.4% of its initial discharge capacity after 1000 cycles [19]. Additionally, biomass-derived hard carbon as anode material for sodium-ion batteries has attracted attention because of its renewable nature and low cost. Qin’s group employed a two-step method to prepare three different structures of hard carbon materials from sisal fibers. It showed the best electrochemical performance, with an initial Coulomb efficiency of 76.7% [20].

Indeed, graphitic carbon nitride (g-C_3_N_4_) is extensively used as an electron transport layer or interfacial buffer layer for realizing photoelectric performance improvement in perovskite solar cells (PSCs). Chu and Li overviewed different g-C_3_N_4_ nanostructures as an additive and surface modifier layers applied to PSCs. They emphasized the mechanism of reducing the defect state in PSCs and proposed the potential challenges and perspectives of g-C_3_N_4_ incorporated into perovskite-based optoelectronic devices [21]. Zhu et al. from Northeastern University investigated the durability of proton exchange membrane fuel cells (PEMFCs) by 300 h accelerated stress test under vibration and non-vibration conditions. The voltage under vibration slightly declines at the current density of 400 mA cm^−2^ and decreases quickly over time in high current density [22].

Hydrogen is regarded as a promising clean energy source in the future due to its high heat value and environmentally friendly features. Sodium borohydride (NaBH_4_) is a good candidate for hydrogen generation from hydrolysis because of its high hydrogen storage capacity and hydrolysis products. However, due to its sluggish hydrogen generation (HG) rate in the water, it usually needs an efficient catalyst to enhance the HG rate. Sun’s group reported graphene oxide (GO)-modified Co-B-P catalysts by a chemical in situ reduction route. The results showed that the as-prepared catalyst with a GO content of 75 mg possesses an optimal catalytic efficiency with an HG rate of 12,087.8 mL min^−1^ g^−1^ at 25 °C [23]. They also synthesized a porous titanium oxide cage (PTOC) using a one-step hydrothermal method using NH_2_-MIL-125 as the template and L-alanine as the coordination agent. Due to the synergistic effect between the PTOC and PtNi alloy particles, the catalysts present a hydrogen generation rate of 10,164.3 mL min^−1^ g^−1^) and activation energy of 28.7 kJ mol^−1^ [24].

Moreover, wearable motion-monitoring systems have been widely studied in recent years. However, traditional wearable devices’ battery energy storage problem limits the development of human sports training applications. Mao and coworkers reported a self-powered, portable micro-structure triboelectric nanogenerator (MS-TENG). It provides a maximum output voltage of 74 V, angular sensitivity of 1.016 V/degree, and high signal-to-noise ratio and can a power electronic calculator and electronic watch. In addition, as a flexible electrode hydrogel, it can readily stretch over 1300%, which can help improve the service life and work stability of MS-TENG [25].
